# Experience of and Worry About Discrimination, Social Media Use, and Depression Among Asians in the United States During the COVID-19 Pandemic: Cross-sectional Survey Study

**DOI:** 10.2196/29024

**Published:** 2021-09-01

**Authors:** Shuya Pan, Chia-chen Yang, Jiun-Yi Tsai, Chenyu Dong

**Affiliations:** 1 School of Journalism and Communication Renmin University of China Beijing China; 2 Center of Journalism and Social Development Renmin University of China Beijing China; 3 School of Educational Foundations, Leadership and Aviation Oklahoma State University Stillwater, OK United States; 4 School of Communication, College of Social & Behavioral Sciences Northern Arizona University Flagstaff, AZ United States

**Keywords:** racial discrimination, experience of discrimination, worry about discrimination, depression, social media use, COVID-19

## Abstract

**Background:**

The COVID-19 outbreak has spurred increasing anti-Asian racism and xenophobia in the United States, which might be detrimental to the psychological well-being of Asian people living in the United States.

**Objective:**

We studied three discrimination-related variables, including (1) experience of discrimination, (2) worry about discrimination, and (3) racism-related social media use during the COVID-19 pandemic among Asians in the United States. We examined how these three variables were related to depression, and how the association between racism-related social media use and depression was moderated by personal experience of and worry about racial discrimination.

**Methods:**

A web-based, cross-sectional survey was conducted. A total of 209 people (mean age 33.69, SD 11.31 years; 96/209, 45.93% female) who identified themselves as Asian and resided in the United States were included in the study.

**Results:**

Experience of discrimination (β=.33, *P*=.001) and racism-related social media use (β=.14, *P*=.045) were positively associated with depressive symptoms. Worry about discrimination (β=.13, *P*=.14) was not associated with depression. Worry about discrimination moderated the relationship between racism-related social media use and depression (β=–.25, *P*=.003) such that a positive relationship was observed among those who had low and medium levels of worry.

**Conclusions:**

The present study provided preliminary evidence that experience of discrimination during the COVID-19 pandemic was a risk factor of depressive symptoms among Asian people in the United States. Meanwhile, racism-related social media use was found to be negatively associated with the well-being of US Asians, and the relationship between social media use and depression was significantly moderated by worry about discrimination. It is critical to develop accessible programs to help US Asians cope with racial discrimination both in real lives and on social media during this unprecedented health crisis, especially among those who have not been mentally prepared for such challenges.

## Introduction

Since the COVID-19 outbreak, anti-Asian racism and xenophobia has been spurred in the United States, and violent attacks against people who appear to be Asian have been documented [1,2]. The growing discrimination against the Asian community, combined with multifaceted pressures from the COVID-19 crisis, can make Asian people more vulnerable to mental health problems. Recent work has provided insights into the deteriorating impact of COVID-fueled discrimination on Asians’ mental health conditions [3,4], but it leaves two important gaps. First, Asians were more likely to use social media for COVID-19–related information than other media sources, thereby amplifying the possibility to encounter discrimination-related news reports or personal narratives [5,6]. However, there has not been sufficient attention toward how using social media to read, post, and talk about discrimination and racism associated with COVID-19 would relate to Asians’ well-being. Second, worry emerges as a common emotional response among these individuals after having directly experienced discrimination and/or reading about personal stories shared on social platforms. Worry could negatively influence one’s mental health, depending on the levels of exposure to racial discrimination. More empirical research is needed to fully explore these contributing factors to mental health outcomes, as Asian ethnic groups face lingering metal health consequences from the COVID-19 pandemic.

In an attempt to empirically examine the psychological consequences of racial discrimination associated with the COVID-19 outbreak among Asian people in the United States, we looked into three factors related to discrimination during this period and examined their associations with depressive symptoms among US Asians. These factors included: (1) experience of discrimination, (2) worry about discrimination, and (3) racism-related social media use during the COVID-19 outbreak. We also investigated whether the relationship between social media use and depression was contingent upon one’s experience of and worry about discrimination.

Racial discrimination is defined as a phenomenon wherein people are unfairly treated or disadvantaged because of their race or phenotypic difference [7]. This could occur in interpersonal interactions, at the workplace, in education settings, or in receiving services and justice [8]. In some worse cases, racial discrimination could take forms of hate crimes or physical attacks [9]. It is not a new phenomenon that the outbreak of an infectious disease could trigger discrimination against racial and ethnic minorities [10]. Several recent studies have documented the salient evidence that Asians in the United States have encountered increasing levels of racial discrimination since the COVID-19 outbreak [11,12].

A huge amount of research has provided empirical evidence that racial discrimination can have negative impacts on the well-being of the stigmatized [13-15]. Racial discrimination can likely damage well-being through the process where one feels being rejected or excluded by the society and thus finds it harder to take control of their lives [16]. The above-described findings were also applicable to the Asian population in the United States. [17-19]. Recent studies have found that racial discrimination against Asian people is negatively associated with psychological well-being among this group during the COVID-19 outbreak [20,21]. One study found that direct experiences of racial discrimination during this period were associated with higher levels of anxiety and depressive symptoms among Chinese American parents and youth [20]. Another study found that vicarious racism, defined as indirect experiences of discrimination experienced by hearing about or seeing racist acts against other members of one’s racial group, was also associated with higher levels of depression and anxiety among Asian Americans [21].

Current conceptualizations of racial discrimination vary across literatures, but almost all attempt to measure it in a subjective way, asking about the perception of racial discrimination in cognitive, affective, and behavioral domains [22]. Some scholars explain that it is because most incidents of discrimination are subtle and hard to document [23]. Importantly, scholars have pointed out the necessity to differentiate two related but distinct constructs of perceived racial discrimination: one is the perception of discrimination actually encountered by oneself and the other is the general worry about the fact that oneself could be a potential target of racial discrimination in the future [24]. It is worthwhile to examine the two constructs’ unique relationship with depression, as previous literature suggested that the two might have different psychological implications. Although experienced discrimination was consistently associated with poor well-being [20,21], the role of worry seemed less conclusive. Worry is a normal mental reaction to impending events in our lives, and it only poses a threat when it becomes persistent and less controllable [25,26]. Under some situations, it can be instrumental by motivating people to cope, to conduct more analytical thinking, and to take more goal-oriented actions [27,28]. In the current study, we included both direct experience of discrimination during the COVID-19 outbreak among our study participants, as well as their worry about being discriminated. Together, we proposed the following hypothesis and research questions:

- Hypothesis 1 (H1): Experience of discrimination would be positively associated with depression among US Asians during the COVID-19 outbreak.
- Research question (RQ) 1: How would worry about discrimination be associated with depression among US Asians during the COVID-19 outbreak?

In addition to the experience of and worry about discrimination, social media also plays a role in US Asians’ psychological well-being during the COVID-19 pandemic [19]. During the pandemic, racism-related content has become more visible on social media. A study found a nearly 10-fold increase of tweets containing “Chinese virus” or “China virus” in the week after former US President Trump mentioned this term on March 16, 2020, compared to the tweets in the week prior [29]. The sentiment analysis of these tweets suggested that the majority contained negative emotions, such as fear, sadness, anger, and disgust [29]. Another study also analyzed the sentiment of race-related tweets before and following the emergence of COVID-19 and found that the proportion of negative tweets referencing Asians increased by 68.4% from November 2019 to March 2020 [30].

Social media use during this period can thus expose Asian users to greater risk for mental health concerns. Incidental and frequent encounter with racist posts on social media might make racism-related information in memory particularly accessible, leading to a perceived social reality where being Asian is unsafe and devalued [31]. A study showed that social media exposure to COVID-related information was associated with higher odds of anxiety and depression among the public in China during the COVID-19 outbreak, after controlling for various demographic factors [32]. The authors attributed the findings to misinformation on social media and users’ risk perception of contracting COVID-19. Although the interpretation is reasonable, for US Asians, engagement with COVID-19–related information on social media, which commonly included anti-Asian sentiments [29,30], may trigger another layer of fear—fear for becoming the target of discrimination or victimization [33]. Nevertheless, to our knowledge, no research has been conducted on how reading, posting, and talking about COVID-19–related racism on social media would relate to US Asians’ mental health. To bridge the gap, we proposed the following hypothesis:

- Hypothesis 2 (H2): Racism-related social media use would be positively related to depression among US Asians during the COVID-19 outbreak.

Finally, this study attempted to examine how the association between racism-related social media use and depression would be moderated by personal experience of and worry about discrimination. As posited in cultivation theory [34,35], the effect of media exposure on personal opinions and feelings would be moderated by personal experiences. They further proposed that the moderation effect could go in two different directions, which were defined as the mainstream effect (ie, people would be more influenced if they have limited experiences of the media content) and the resonance effect (ie, people would be more influenced if they have more experiences of the media content).

The vulnerability-stress model also argues that the extent to which a stressful event could trigger a mental disorder would depend on an individual’s vulnerability, which could be psychological, biological, or situational factors [36]. We considered depression as the result of the interaction between worry about discrimination and racism-related social media use. As mentioned above, worry is an emotional reaction to future uncertainties, which could play a positive role in problem-solving in some situations [27,28]. In our case, worry might buffer the influence of racism-related social media use on depression by motivating people to solve the problem. Meanwhile, it is also possible for worry to play a negative role in such relationships if it does not direct people to take positive actions. As the moderating roles of discrimination-related experience and worry remained unclear, we proposed the following two additional research questions:

- RQ2: How would the experience of discrimination moderate the relationship between social media use and depression among US Asians during the COVID-19 outbreak?
- RQ3: How would worry about discrimination moderate the relationship between social media use and depression among US Asians during the COVID-19 outbreak?

## Methods

### Procedures

A web-based, cross-sectional survey approved by the institutional review board was first created on Qualtrics and then distributed to qualified participants via Amazon Mechanical Turk (MTurk) in mid-May 2020. Eligible participants included those belonging to Asian ethnic groups and residing in the United States. The first page of the questionnaire was a consent form providing the participants with brief background on the study and information about anonymity, confidentiality, and compensation. The rest of the pages asked questions about demographic information, media usage, discrimination-related experiences and perceptions during the COVID-19 outbreak, and some psychological measurements. Attention checkers were included to ensure data quality. The survey took an average of 15 minutes to complete.

### Participants

We recruited 242 eligible participants who were Asians residing in the United States, and a total of 209 participants were included in this study after using a listwise deletion approach (ie, those who answered “not sure” or “prefer not to answer” in any questions were coded as having a missing value and were excluded from the analyses). To determine if we achieved the needed sample size for our regression analyses, an a priori sample size calculator for multiple regressions was used. The result showed that, with the anticipated medium effect size set at 0.15, the statistical power level set at 0.80, and the significance level set at *P*=.05, the minimum sample size required for the current study was 131. Thus, we had reached the sample size requirement in this study.

The participants were between the ages of 18 and 73 years (mean 33.69, SD 11.31 years). Among them, 96 (45.93%) were female and 113 (54.07%) were male participants. Most participants were either single (n=105, 50.24%) or married (n=97, 46.41%). The majority of the participants had a bachelor’s degree (n*=*106, 50.72%), with the remaining holding a degree below (n*=*50, 23.92%) or above the bachelor’s level (n*=*53, 25.36%). About half of the participants (n=97, 46.41%) has a full-time employment. The income levels were relatively evenly distributed among participants, but more than half of the participants (n=115, 55.02%) reported that their income had somehow been affected by the COVID-19 pandemic. Meanwhile, most participants did not identify themselves as Chinese (n=135, 64.59%). Table 1 presents demographic information of the sample.

**Table 1:** Descriptive analysis of the sample (N=209).

Variables			Respondents, n (%)
**Age in years (range: 18-73 years)**			
	≤30	92 (44.02)	
	>30	117 (55.98)	
**Gender**			
	Male	113 (54.07)	
	Female	96 (45.93)	
**Marital status**			
	Single	105 (50.24)	
	Married	97 (46.41)	
	Widowed	2 (0.96)	
	Divorced	5 (2.39)	
**Education**			
	Less than high school degree	1 (0.48)	
	High school graduate	8 (3.83)	
	Some college but no degree	29 (13.88)	
	Associate degree	12 (5.74)	
	Bachelor’s degree	106 (50.72)	
	Master’s degree	39 (18.66)	
	Doctoral degree	14 (6.70)	
**Employment status**			
	Employed, working 40 or more hours per week	97 (46.41)	
	Employed, working 1-39 hours per week	46 (22.01)	
	Not employed, looking for work	41 (19.62)	
	Not employed, *not* looking for work	21 (10.05)	
	Retired	4 (1.91)	
**Income (US $)**			
	<10,000	15 (7.18)	
	10,000-19,999	12 (5.74)	
	20,000-29,999	12 (5.74)	
	30,000-39,999	13 (6.22)	
	40,000-49,999	18 (8.61)	
	50,000-59,999	18 (8.61)	
	60,000-69,999	11 (5.26)	
	70,000-79,999	19 (9.09)	
	80,000-89,999	15 (7.18)	
	90,000-99,999	17 (8.13)	
	100,000-149,999	37 (17.70)	
	≥150,000	22 (10.53)	
**Income affected**			
	Yes	115 (55.02)	
	No	94 (44.98)	
**Chinese**			
	Yes	74 (35.41)	
	No	135 (64.59)	

### Measurements

#### Experience of Discrimination

The 5-item Everyday Discrimination Scale [37] was adapted to measure how often the respondents had experienced discrimination since the COVID-19 outbreak. The scale ranged from 1 (“never”) to 5 (“a lot”). The five items were as follows: (1) “You are treated with less courtesy or respect than other people”; (2) “You receive poorer service than other people”; (3) “People will act as if they think you are dangerous”; (4) “People act as if they are afraid of you”; (5) “You are threatened or harassed.” The item “People act as if they think you are not smart” from the original scale was changed as “People act as if you are dangerous” to better indicate the common bias against Asians during the COVID-19 pandemic. Cronbach α for this scale was .94 (mean score 9.60, SD 4.78).

#### Worry About Discrimination

Participants were also asked to rate the extent to which they worry about the listed things in Everyday Discrimination Scale [37]. The items were changed to future tense since worry tends to be an emotion related to things that may happen in the future. Cronbach α for this scale was .96 (mean score 12.96, SD 5.91).

#### Racism-Related Social Media Use

We created a 4-item scale to measure the extent to which the participants used social media to read, post, and talk about racism-related information associated with COVID-19 since the outbreak of the pandemic. The 4 items on this scale were as follows: (1) “On social media, how much do you pay attention to and read about racism related to COVID-19?” (2) “On social media, how much do you post or repost information and news about racism related to COVID-19?” (3) “On social media, how much do you discuss racism related to COVID-19 with others through commenting?” and (4) “On social media, how much do you discuss racism related to COVID-19 with others through private messaging?” A 7-point Likert scale, ranging from 1 (“not at all”) to 7 (“very much”), was used. Cronbach α for this scale was .85 (mean score 12.76, SD 6.40).

#### Depression

A 10-item depression scale [38] was adopted to measure the level of depression among US Asians. Respondents were asked to rate how often they felt they “[C]ouldn’t shake off the blues even with help from your family and your friends,” for example. The scale ranged from 1 (“rarely or none of the time”) to 4 (“most or all of the time”). After performing confirmative factor analysis, three reverse-coded items were removed (see details below). Cronbach α for this 7-item scale was .88 (mean score 21.51, SD 5.97).

#### Control Variables

Respondents reported basic demographic information, including age, gender, marital status, education level, employment status, and income. In addition, self-identification as Chinese was also considered as a control factor because of the close connection between the COVID-19 outbreak and China. We also asked respondents whether their family incomes had been affected since the COVID-19 outbreak, as it has been documented as a key stressor during the pandemic [39].

### Data Analysis

To address the issues of measurement errors, we performed confirmatory factor analysis in Mplus (version 7; Muthén & Muthén) by considering experience of discrimination, worry about discrimination, social media use, and depression as *latent variables*. The initial model fit was mediocre, and 3 reverse-coded items of the depression scale had low loadings (<0.40). After removing these 3 items, the measurement model fit was acceptable (comparative fit index=0.92, Tucker–Lewis index=0.91, root mean square error of approximation= 0.08).

We then used SPSS software (version 26.0; IBM Corp) to perform a 3-step hierarchical regression analysis. The first model included *control variables*. We dichotomized gender, marital status, employment status, and self-identification as Chinese, with those who were male, married, employed full-time, and self-identified as Chinese being coded as “1,” and the other categories coded as “0.” In the second model, the following 3 key independent variables were added: (1) experience of discrimination, (2) worry about discrimination, and (3) racism-related social media use. These variables were mean-centered. In the third model, the two interaction terms examining the moderating roles of experience of discrimination and worry about discrimination were added. No collinearity problems were identified after the mean-centering approach. The significance level was set at *P*<.05.

## Results

As shown in Table 2, all three regression models were significant: model 1 (R^2^=0.11, *F*_8,200_= 3.07; *P*=.003), model 2 (R^2^=0.35, *F*_11,197_= 9.71; *P*<.001), and model 3 (R^2^=0.38, *F*_13,195_= 9.21; *P*<.001). Model 2 with three discrimination-related variables significantly increased the explaining power for depression (R^2^ change=0.24; *F* change=24.51; *P*<.001).

Experience of discrimination was positively associated with depressive symptoms (β=.33, *P*=.001), supporting hypothesis 1. Racism-related social media use (β=.14, *P*=.045) was also positively related to depression, supporting hypothesis 2. Worry about discrimination (β=.13, *P*=.14) was not associated with depression (RQ1).

**Figure 1:**
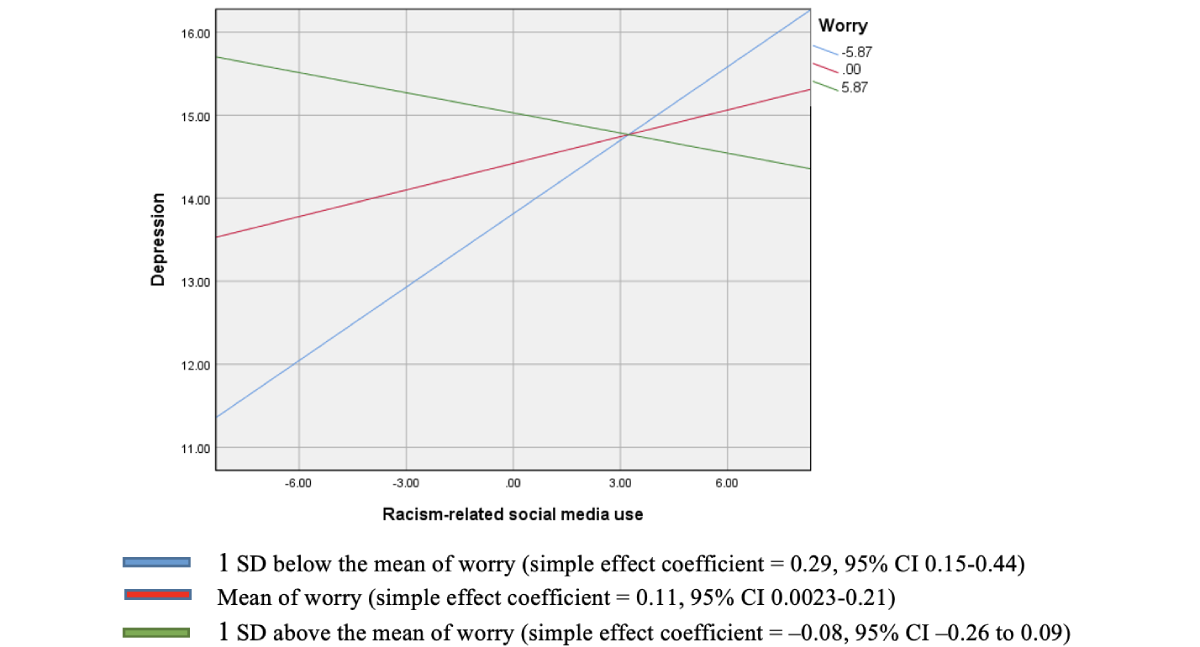
Interactive plot showing predicted values of depression as the function of racism-related social media use and worry about discrimination.

To answer RQ2 and RQ3, we found the significant interactive effect of social media use and worry about discrimination on depression (β=–.25, *P*=.003). To visualize the moderation effect, we used Process version 3.5 to conduct simple slope analysis and produce the figure of the interaction. As depicted in Figure 1, among participants with low (1 SD below the mean) and medium levels of worry, social media use was positively and significantly related to depression (simple effect coefficient=0.29 for low worry, 95% CI 0.15-0.44; simple slope coefficient=0.11 for medium worry, 95% CI 0.0023-0.21). No significant relationship between social media use and depression was found at a high level of worry.

In addition, female Asians were found to be more depressed than male Asians (β=–.14, *P*=.02), and Chinese Asians were less depressed than those Asians who were not Chinese (β=–.12, *P*=.045).

**Table 2:** Ordinary least squares multiple regression analysis of depression scores as a function of discrimination-related variables and control variables (N=209).

Variables	Model 1		Model 2		Model 3	
	β	*P* value	β	*P* value	β	*P* value
Age	–.18	.02	–.15	.029	–.13	.05
Gender (male)	–.12	.08	–.14	.020	–.14	.02
Marital status (married)	–.08	.29	–.10	.16	–.11	.12
Education	–.04	.62	–.06	.33	–.07	.27
Employed (full time)	.04	.62	.03	.61	.02	.72
Income	–.06	.45	–.04	.52	–.06	.37
Income affected (yes)	.12	.07	.07	.28	.06	.31
Chinese	.00	.99	–.11	.06	–.12	.045
Experience of discrimination	N/A^a^	N/A	.34	<.001	.33	.001
Worry about discrimination	N/A	N/A	.07	.40	.13	.14
Social media use	N/A	N/A	.18	.012	.14	.045
SM^b^×Experience^c^	N/A	N/A	N/A	N/A	.16	.10
SM×Worry^d^	N/A	N/A	N/A	N/A	–.25	.003

^a^N/A: not applicable.

^b^SM: racism-related social media use.

^c^Experience: experience of discrimination.

^d^Worry: worry about discrimination.

## Discussion

### Principal Findings

The present study provides preliminary evidence that experience of discrimination during the COVID-19 pandemic is a risk factor for depressive symptoms among Asians in the United States. Meanwhile, racism-related social media use was also found to be negatively related to the well-being of US Asians, and the relationship between social media use and depression was moderated by worry about discrimination, such that social media use was associated with greater depression among those who were less worried about discrimination.

Drawing on survey data from 209 Asians residing in the United States, the study presents the findings consistent with earlier research [20,21] showing that experience of racial discrimination induced by the COVID-19 pandemic was negatively related to the well-being among Asian people. Effective interventions should be designed to provide timely assistance to Asians who have experienced discrimination during the pandemic to prevent them from developing further depressive symptoms. One limitation of the current study is the use of an existing measurement of experienced racial discrimination, which may fail to capture the specific experiences of racial discrimination associated with the COVID-19 pandemic. Further research could focus on understanding the unique elements of racial discrimination in the COVID-19 context so that more accurate conclusions could be made.

Using social media to read, post, and talk about racism was also found to be associated with higher depression. It is noteworthy that the relationship was significant when controlling for direct experience of discrimination (albeit with a smaller effect size than direct experience). Given how fast-growing and visible anti-Asian sentiments have been on social media during the pandemic [29,30], and that Asians were more likely to use social media than other media sources to obtain COVID-19–related information [5,6], our finding deserves serious attention. It is also worth mentioning that our social media use scale includes a variety of social media activities (eg, reading, posting, commenting, private messaging) through which individuals may be exposed to and engage with racism-related information. Relative to single-item questions asking about participants’ social media use in one particular way, such as exposure to or consumption of a certain type of information [32,40], our scale has the strength of more comprehensively capturing multiple ways of social media use during the pandemic.

Worry about discrimination was not directly associated with depression in the present study. When worry about discrimination was considered alongside the actual experience of discrimination, it was the actual experience that was detrimental. As shown in previous literature, the benefits and detriments of worry may have cancelled out each other, leading to a nonsignificant direct association [25-28]. It may be worthwhile for future research to explore possible moderators of this relationship, such as coping styles and social support-seeking behaviors, to better understand the implications of this type of worry for mental health.

Although worry was not a significant independent variable, it was a significant moderator for the relationship between social media use and depression. Specifically, among Asians with low and medium levels of worry about discrimination, social media use was related to higher levels of depression. A possible explanation for our finding might be that Asians with low to medium worry were less prepared for anti-Asian discrimination. Thus, they felt more overwhelmed and depressed when exposed to greater amount of racism-related information on social media. Future research should explore in detail how US Asians with low and high worry cope with racial discrimination in different ways that may buffer or exacerbate the influence of social media use on depression. Meanwhile, it is also worthwhile to explore more factors that might moderate the relationship between social media use and depression, such as coping strategies, perceived social support, and so forth.

In terms of demographic factors considered in our regression model, we found that female Asians were more depressed than male Asians, which is consistent with findings of previous studies [41,42]. Surprisingly, Chinese Asians were found to be less depressed than Asians who were not Chinese. Although Asians are normally being treated as one single ethnic group in the United States, research does indicate that there might be differences in the mental health mechanisms among various Asian ethnicities. For example, it was indicated that Chinese Americans had lower prevalence of depression than Korean and Vietnamese Americans [43]. Further study is needed to identify how the mental health conditions of different Asian groups have been impacted by the COVID-19 pandemic.

### Limitations and Conclusions

The study has some limitations. The first is that the research model was not evaluated with a random sample. Therefore, readers are advised to take caution when generalizing the results to the overall Asian population in the United States or Asian groups in another country. Another limitation is that the cross-sectional study does not allow for drawing causal conclusions, and thus the findings should be interpreted as correlations rather than as having a cause-and-effect relationship.

Despite all these limitations, the present study extends the current literature on rising anti-Asian discrimination and associated mental health outcomes in several ways. First, we differentiated the actual experience of discrimination and worry about discrimination and revealed their unique relationship with depression among US Asians in the context of the COVID-19 pandemic. Another theoretical contribution is providing important insights into the specific role of racism-related social media use in mental health among a minority group. Importantly, the results suggest that US Asians with higher versus lower levels of worry face different degrees of susceptibility to the negative influences of racism-related social media use on mental health.

From a practical perspective, immediate and effective interventions should be implemented to decrease racist and discriminatory behaviors against US Asians, as direct experience of discrimination was found to be a robust risk factor for depression among this group, after controlling for various demographic variables. It is also critical to develop accessible programs to help US Asians cope with racial discrimination both in real lives and on social media during this unprecedented health crisis, especially among those who have not been mentally prepared for such challenges.

## Abbreviations

H1: hypothesis 1
H2: hypothesis 2
RQ: research question
